# Monocyte-derived IL-1β predicts and promotes HBsAg decline in chronic hepatitis B patients under nucleoside analogue therapy

**DOI:** 10.1097/HC9.0000000000000957

**Published:** 2026-05-26

**Authors:** Satoshi Shigeno, Takahiro Kodama, Kazuhiro Murai, Emi Sometani, Kentaro Yasuda, Yuki Tahata, Akira Nishio, Satoshi Tanaka, Masanori Miyazaki, Kazuyoshi Ohkawa, Naruyasu Kakita, Seiichi Tawara, Takayuki Yakushijin, Hayato Hikita, Hidenori Toyoda, Tetsuo Takehara

**Affiliations:** 1 Department of Gastroenterology and Hepatology, The University of Osaka, Graduate School of Medicine, Suita, Japan; 2 Department of Gastroenterology and Hepatology, Japan Community Healthcare Organization Osaka Hospital, Osaka, Japan; 3 Department of Gastroenterology and Hepatology, National Hospital Organization Osaka National Hospital, Osaka, Japan; 4 Department of Gastroenterology and Hepatology, Osaka Police Hospital, Osaka, Japan; 5 Department of Hepatobiliary and Pancreatic Oncology, Osaka International Cancer Institute, Osaka, Japan; 6 Department of Gastroenterology and Hepatology, Kaizuka City Hospital, Osaka, Japan; 7 Department of Gastroenterology and Hepatology, Osaka General Medical Center, Osaka, Japan; 8 Department of Gastroenterology, Ogaki Municipal Hospital, Ogaki, Japan; 9 Department of Gastroenterology and Hepatology, Kansai Rosai Hospital, Amagasaki, Japan

**Keywords:** antigen presentation, biomarker, chronic hepatitis B, functional cure, hepatitis B surface antigen, IL-1β, innate immunity, monocytes, nucleos(t)ide analog, single-cell RNA sequencing

## Abstract

**Background:**

Although nucleos(t)ide analogs (NAs) suppress HBV replication, functional cure—defined by hepatitis B surface antigen (HBsAg) loss—is rarely achieved in chronic hepatitis B (CHB). Immune correlates of HBsAg decline may inform new therapeutic strategies.

**Methods:**

We profiled peripheral blood mononuclear cells (PBMCs) from NA-treated CHB patients with declining versus sustained HBsAg levels by single-cell RNA sequencing (scRNA-seq), followed by validation in an independent multicenter cohort using transcriptomic and functional assays. In a retrospective cohort, we evaluated serum interleukin-1β (IL-1β) as a predictor of HBsAg decline during long-term follow-up.

**Results:**

scRNA-seq revealed that CD14^+^CD16^−^ classical monocytes in the HBsAg-declining group expressed significantly higher levels of IL-1β, along with genes involved in antigen presentation and phagocytosis. In the validation cohort, IL-1β expression in circulating CD14^+^ classical monocytes was also significantly higher in the declining group. Ex vivo stimulation assays demonstrated that recombinant HBsAg induced IL-1β production in monocytes, with stronger responses in the declining group. Recombinant IL-1β suppressed HBsAg secretion from HBV-producing HepG2.2.15 cells in vitro in a dose-dependent manner. In a longitudinal cohort, elevated serum IL-1β predicted subsequent HBsAg decline with an AUROC of 0.71. Multivariate analysis confirmed IL-1β as an independent predictor.

**Conclusions:**

Monocyte-derived IL-1β is linked to HBsAg decline during NA therapy and may serve as both a predictive biomarker and a potential therapeutic target to achieve functional cure.

## Graphical abstract

**Figure:**
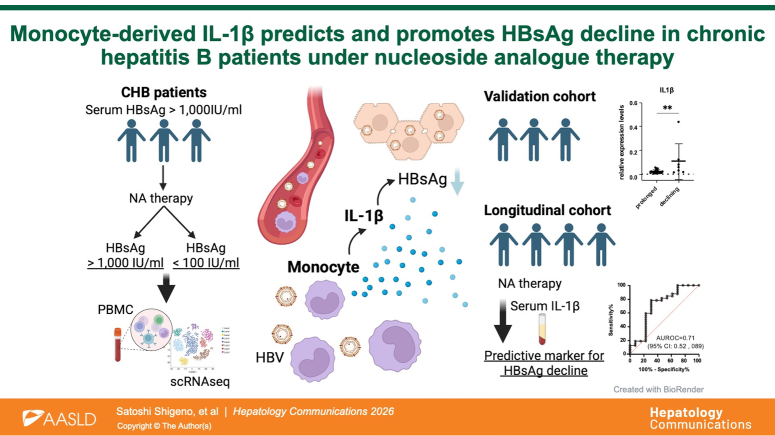

## INTRODUCTION

Hepatitis B virus (HBV) infection remains one of the world’s most prevalent—and deadly—infectious diseases. The WHO Global Hepatitis Report 2024 estimates that ~254 million people (about 3% of the global population) were living with chronic HBV infection in 2022, and some 1.1 million people died from its sequelae, mainly cirrhosis and hepatocellular carcinoma. Despite a safe, highly effective vaccine and potent nucleos(t)ide-analogue (NA) therapies, worldwide only 13% of infected individuals are diagnosed and barely 3% receive treatment, still below the levels needed to reach the WHO’s 2030 elimination targets.^1^

NAs such as entecavir or tenofovir suppress serum HBV DNA to below the limit of quantification in >90% of treatment-naïve adults and normalize alanine aminotransferase (ALT) in roughly three-quarters within the first 48–96 weeks, achieving the primary short-term therapeutic benchmarks for chronic hepatitis B (CHB).^2^,^3^ Despite continued therapy, a durable “functional cure” is uncommon: long-term cohort analyses document cumulative HBsAg loss of only 1%–3% after 5–8 years of uninterrupted NA exposure.^4^,^5^ The key barrier is the persistence of nuclear covalently closed circular DNA (cccDNA) and integrated viral sequences, which remain detectable even under profound viremic suppression and serve as templates for renewed replication.^6^,^7^ Accordingly, the 5-year cumulative HCC incidence was 5.8% at 10 years after NA initiation,^8^ while withdrawal studies show virological relapse in 40%–60% of patients within 1 year of stopping therapy.^9^ These limitations have catalyzed an active development pipeline of finite, drug-free regimens—HBsAg-targeted RNA interference or antisense oligonucleotides, capsid-assembly modulators, entry inhibitors, and immune-directed combinations—aimed at eliminating or permanently silencing cccDNA to deliver a durable cure beyond the reach of current NAs.^10^,^11^

Studies of spontaneous viral clearance and acute hepatitis B have demonstrated that, in addition to adaptive immune responses mediated by HBV-specific T and B lymphocytes, innate immunity—particularly natural killer (NK) cells—plays a pivotal role in eliminating HBV.^12^,^13^ In CHB, however, these immune mechanisms are functionally compromised. Accumulating evidence indicates that HBV modulates the production of chemokines and the expression of ligand–receptor pairs within hepatocytes, thereby subverting both innate and adaptive immunity.^14^,^15^ Therapeutic interventions that disrupt these viral immune-evasion pathways hold promise for achieving true viral eradication and would constitute a paradigm shift beyond the capabilities of existing treatments.

Monocytes act as a pivotal bridge between the innate and adaptive immune systems. As circulating sentinels of the innate arm, they sense pathogens via pattern-recognition receptors, rapidly secrete cytokines/chemokines, and phagocytose microbes. Upon tissue entry, they differentiate into monocyte-derived macrophages or dendritic cells that upregulate MHC-II and costimulatory molecules, enabling them to present processed antigen to naïve or memory T cells and to polarize T-cell responses through soluble mediators.^16^,^17^

The growing application of single-cell transcriptomics has enabled detailed gene-expression profiling of host immune responses across the clinical phases of hepatitis B in human samples^18^ and produced a handful of reports on the rare individuals who achieve complete HBsAg seroclearance.^19^ Yet comprehensive single-cell interrogation of the immune landscape in patients undergoing the critical HBsAg-decline phase—a pivotal step toward functional cure—remains largely absent, and the role of monocytes in this trajectory is still poorly defined. This study aimed to dissect peripheral immune dynamics at single-cell resolution in CHB patients who exhibited marked HBsAg declines, with the goal of identifying immune factors linked to HBsAg reduction and uncovering novel therapeutic targets and predictive biomarkers.

## METHODS

### Study design

The first cohort of the present study recruited patients with CHB from the University of Osaka Hospital. We first identified CHB patients (HBsAg ≥1000 IU/mL at pre-treatment) who had received NA therapy for more than 2 years and achieved undetectable HBV DNA after treatment. Patients with baseline HBsAg levels ≤1000 IU/mL were excluded to minimize inclusion of individuals with a higher likelihood of spontaneous HBsAg decline.^20^ We then selected 4 patients whose HBsAg levels fell below 100 IU/mL (“declining” group) during long-term NA therapy and 4 whose HBsAg levels remained above 1000 IU/mL (“prolonged” group). The other inclusion criteria included age <70 years at the time of sample collection and treatment duration <15 years. The exclusion criteria included patients with advanced liver fibrosis or cirrhosis, serious other organ disease, use of immunosuppressive agents, and coexistence of malignant tumors. We collected PBMCs from these eight patients at the enrolment of this study with written informed consent.

For the validation cohort, additional 31 CHB patients (“declining group”: n=24, “prolonged group”: n=7) satisfied with above criteria were prospectively recruited from NHO Osaka National Hospital (Osaka, Japan), Osaka Police Hospital (Osaka, Japan), The University of Osaka Hospital, Osaka International Cancer Institute (Osaka, Japan), Kaizuka City Hospital (Osaka, Japan), and Osaka General Medical Center (Osaka, Japan). PBMCs were collected at the enrolment of this study with the written informed consent.

In the long-term follow-up cohort, 45 CHB patients from Ogaki Municipal Hospital (Ogaki, Japan) receiving NA treatment for at least 1 year and showing HBsAg levels above 1000 IU/mL at the time of serum collection were enrolled. Based on the serum HBsAg levels after more than 3 years of NA treatment, patients were classified into “declining” (n=13) and “prolonged” (n=32) groups.

### Ethics approval

This study complied with the Declaration of Helsinki and Istanbul, the Ethical Guidelines for Clinical Studies of the Japanese Ministry of Health, Labor and Welfare, and all applicable Japanese regulations. Written informed consent was obtained from all patients. The protocol was approved by the Ethics Review Committee of The University of Osaka Hospital (approval No. 12238) and by the institutional review board of each participating center.

### Single-cell preparation

For peripheral blood mononuclear cells (PBMCs) isolation, 10 mL of heparinized blood was diluted 1:1 with PBS, gently layered over an equal volume of lymphocyte separation medium (Nacalai Tesque, Japan), and centrifuged at 740*g* for 30 minutes; the mononuclear cell layer was then harvested.

### Library preparation and sequencing

Isolated PBMCs were freshly subjected to single-cell RNA sequencing using the BD Rhapsody platform (BD Biosciences). Cell capture and library preparation were performed with the BD Rhapsody Human Immune Response Targeted Panel, including 397 immune-related genes, following the manufacturer’s instructions, and libraries were sequenced on an MGI DNBSEQ-G400RS.

### Single-cell RNA sequencing (scRNA-seq)

FASTQ files were processed with the BD Rhapsody Targeted Analysis Pipeline (Revision 10) on the Seven Bridges platform, and molecule-count matrices were further analyzed in SeqGeq (v1.8.0). Quality control followed the standard SeqGeq workflow, including evaluation of a knee‑calling plot, total UMI counts, and gene numbers to exclude potential doublets, empty wells, and low‑quality cells. Dimensionality reduction (UMAP) and clustering were carried out in Seurat (v4.3.0) with the SCTransform workflow for normalization and variance stabilization. Clustering was iterated at resolutions from 0.2 to 2.0 in 0.2 increments. Cell identities were then assigned manually on the basis of canonical marker-gene expression. For downstream analyses, cluster-level expression matrices were exported from SeqGeq and processed in R. Gene-set enrichment analysis was performed with clusterProfiler (v4.4.4) and trajectory inference with Monocle 3 (v1.2.7). Functional module scores were calculated as the *z*-score–normalized mean expression of constituent genes, and enrichment scores were obtained with GSEA.

### mRNA expression measurement

Heparinized blood samples obtained at the participating center were kept fresh and transported to the laboratory on the day of collection. PBMCs were isolated as described above, and CD14^+^ monocytes were subsequently purified by positive selection with CD14 MicroBeads (Miltenyi Biotec, Germany). Total RNA isolated from CD14^+^ monocytes using RNeasy Mini Kit (#74104; Qiagen) was reverse-transcribed, and the mRNA expression levels were quantified using TaqMan Gene Expression Assays (Thermo Fisher Scientific, MA, USA; Humanβ-actin, Hs01060665_g1; Human IL-1β, Hs01555410_g1).

### Ex vivo stimulation assay

Two million PBMCs isolated as described above were cultured for 6 hours in 500 µL of RPMI-1640 medium (Thermo Fisher Scientific) containing Brefeldin A (GolgiPlug, 1 µL/mL) and supplemented with either recombinant HBsAg at 3 µg/mL or 30 µg/mL, or lipopolysaccharide (LPS) at 100 ng/mL. Cells adherent to the wells were detached by incubating with 2 mM EDTA at 4 °C for 10 minutes and were subsequently used for the flow cytometry analyses.

### Intracellular cytokine staining and flow cytometric analysis

The IL-1β expression of CD14^+^ monocytes was evaluated using intracellular cytokine staining. After staining with mAb of CD3 (BD #555332), CD19 (BD #555412), CD56 (BD #562794), CD14 (BD #566466) and CD16 (BD #5638289) for 15 minutes, the cells were fixed and permeabilized with fixation/permeabilization solution (BD #554715) for 20 minutes at room temperature and stained with IL-1β (BioLegend #508207). In this flow cytometry analysis, dead-cell staining was not performed in order to minimize assay time. The stained cells were acquired on a FACS Canto II (Becton Dickinson, NJ, USA). The data were analyzed using FlowJo (Becton Dickinson).

### In vitro stimulation assay

The HBV-producing cell line HepG2.2.15 (1×10^5^ cells) was seeded into collagen-coated wells and treated with recombinant IL-1β (Abcam, #91276) at 0, 10, or 100 ng/mL. HepG2.2.15 cells were harvested at confluence at the time of supernatant collection. HBsAg levels in the culture supernatant were then measured by a standard method at SRL, Inc. (Tokyo, Japan).

### Serum cytokine detection

Serum cytokine levels were evaluated using the ProQuantum Immunoassay Kit (Thermo Fisher Scientific, A35574), a highly sensitive protein quantitation assay combining the analyte specificity of antibody antigen binding with the signal detection and amplification performance of real-time PCR, following the manufacturer’s instructions. The cytokine concentrations were determined using the calculated standard curve.

### Statistical analysis

All data are expressed as the mean ± SD. To assess the significant differences between the 2 groups, 2-tailed Student *t* test was performed. A *p*-value <0.05 was considered to indicate statistical significance. One-way ANOVA with the Tukey multiple comparisons test was used to compare more than 2 groups. To assess the predictive value of HBsAg decline, we generated a receiver operating characteristic (ROC) curve and calculated the area under the curve (AUC). Variables incorporated into the multivariate logistic regression model were selected on the basis of their potential as confounders, the magnitude of their odds ratios, and their *p* values in the univariate analyses. Given the limited number of outcome events (n = 13), the number of covariates included in the multivariable logistic regression model was restricted to avoid overfitting. The test in the logistic regression analysis was performed using the likelihood ratio test. Statistical analysis was performed with GraphPad Prism 9 v9.4.1 (GraphPad Software, LLC., CA, USA) and JMP Pro 17.2.0. (SAS Institute, Inc., Tokyo, Japan).

## RESULTS

### Immune profiling of PBMCs in NA-treated CHB patients with differential HBsAg dynamics

**FIGURE 1:**
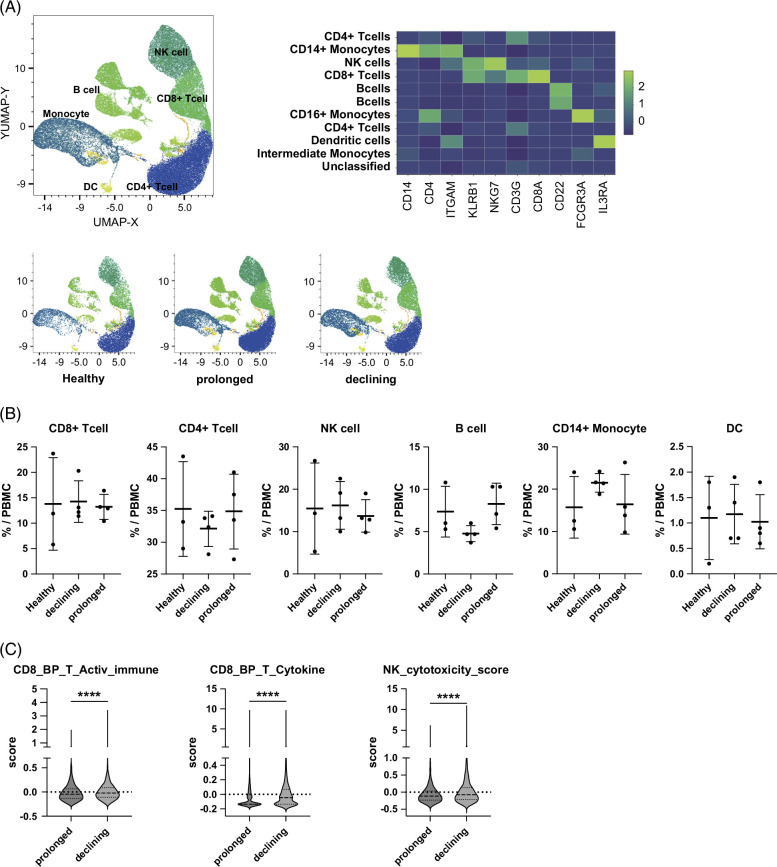
Activated immune response may be associated with HBsAg decline in CHB patients under NA treatment. (A–C) Peripheral blood mononuclear cells from CHB patients and healthy controls were analyzed by scRNA-seq (n = 4 for each of the declining group and prolonged group, n = 3 for healthy controls). (A) A total of 81,657 cells were classified into 11 major clusters described by UMAP (upper left). Heatmap showing the expression levels of representative genes for each cell type (upper right). The distribution of each group on the UMAP (bottom). (B) The population of each major cluster. (C) Violin plots showing GO term scores for “T-cell activation involved in immune response”, “T-cell cytokine production”, and “NK-cell cytotoxicity”. ****p<0.0001. Abbreviations: CHB, chronic hepatitis B; GO, gene ontology; HBsAg, hepatitis B surface antigen; NA, nucleos(t)ide analogue; NK, natural killer; PBMCs, peripheral blood mononuclear cells; scRNA-seq, single-cell RNA sequencing; UMAP, uniform manifold approximation and projection.

To identify the immunological factors regulating HBsAg levels under NA treatment, we explored the host immunodynamics by scRNA-seq of PBMCs in CHB patients. Among CHB patients with high HBsAg levels before the NA treatment (>1000 IU/mL) treated with more than 2 years of NA treatment, we selected 4 patients whose HBsAg levels decreased below 100 IU/mL (declining group) and 4 patients whose HBsAg levels remained above 1000 IU/mL (prolonged group). Together with 3 healthy controls, we performed scRNA-seq of PBMCs targeting the 397 immune-related genes. The average duration of NA treatment for the 8 CHB patients was 9.5 years, and there were no differences in clinical backgrounds, including age, gender, type of NA treatment, or duration of treatment between the declining and prolonged groups (Supplemental Table S1, http://links.lww.com/HC9/C340). Two participants of each group had a flare history defined as an elevation of ALT to ≥5 times the upper limit of normal. A total of 81,657 PBMCs were categorized into 10 immune cell clusters, including CD8^+^ T cells, CD4^+^ T cells, NK cells, B cells, monocytes, and dendritic cells, based on the expression of characteristic marker genes (Figure 1A). The frequency of each cell population was not different between the declining and prolonged groups (Figure 1B). Gene-set scoring demonstrated that CD8^+^ T-cell clusters and NK clusters in the HBsAg-declining group were significantly elevated for the GO term scores for “T-cell activation involved in immune response”, “T-cell cytokine production”, and “NK cytotoxicity” (Figure 1C). Collectively, these findings indicate that activation of both innate and adaptive immunity is linked to HBsAg decline in NA-treated CHB patients.

### Increased IL-1β production by CD14^+^ classical monocytes is associated with HBsAg decline in NA-treated CHB patients

**FIGURE 2:**
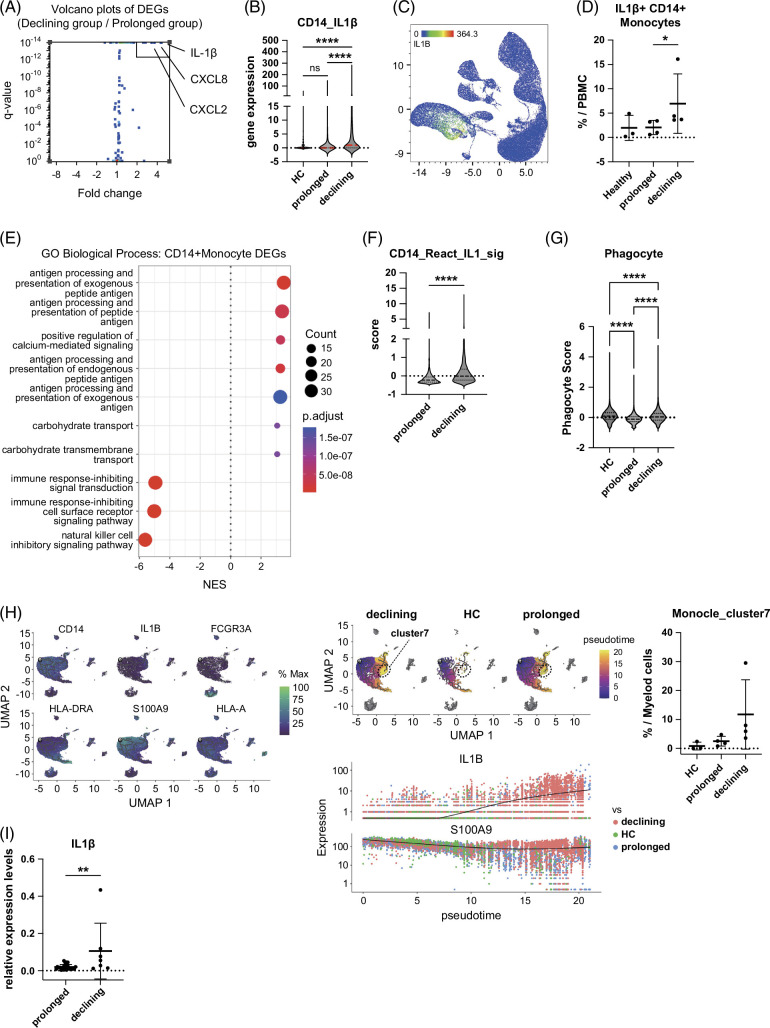
High IL-1β production from the CD14^+^CD16^−^ classical monocytes may be associated with HBsAg decline in CHB patients under NA treatment. (A) Volcano plot of differential gene expression of CD14^+^ monocytes (declining group vs. prolonged group). (B) The IL-1β expression levels of the CD14^+^CD16^−^ classical monocyte cluster in the indicated group (HC: healthy control). (C) The distribution of IL-1β–expressing cells in the PBMCs. (D) The population of IL-1β^+^CD14^+^CD16^−^ classical monocyte of each group. (E) Gene enrichment analysis (Gene Ontology, GO) showed the top 10 pathways. (F, G) Violin plots showing Reactome Interleukin-1 signaling score and the phagocyte score. (H) Pseudotime trajectory analysis of myeloid cells, including CD14^+^ monocytes. (I). IL-1β mRNA expression of isolated circulating CD14^+^ monocytes between the declining group versus the prolonged group among 31 CHB patients. *p<0.05. Abbreviations: CHB, chronic hepatitis B; DEGs, differentially expressed genes; GO, Gene Ontology; HC, healthy control; HBsAg, hepatitis B surface antigen; IL-1β, interleukin-1 beta; mRNA, messenger RNA; NA, nucleos(t)ide analogue; NES, normalized enrichment score; PBMCs, peripheral blood mononuclear cells.

We then analyzed the differentially expressed genes (DEGs) of each cluster. Notably, the CD14^+^CD16^−^ classical monocyte subset showed pronounced differential expression of genes such as IL-1β, CXCL8, and CXCL2 (Figure 2A). The IL-1β expression levels of the CD14^+^CD16^−^ classical monocyte cluster were significantly higher in the declining group than those in the prolonged group and the healthy control group (Figure 2B). The IL-1β expression was mostly restricted to the CD14^+^CD16^−^ classical monocyte cluster in the PBMC (Figure 2C). In addition, the number of IL-1β^+^CD14^+^CD16^−^ classical monocytes was significantly higher in the declining group than in the prolonged group and the healthy control group (Figure 2D). Gene set enrichment analysis (GSEA) revealed the positive enrichment scores for multiple pathways related to antigen presentation in the declining groups compared with those in the prolonged groups, suggesting the activation of these pathways (Figure 2E). The Reactome Interleukin-1 signaling score and the phagocyte score were elevated in the CD14^+^ monocytes in the declining groups (Figures 2F, G). Using the Monocle pseudotime algorithm, we reconstructed myeloid trajectories that include CD14^+^ monocytes and observed a progressive rise in IL-1β expression from the S100A9-high immature root, with a differentiation bias evident in the HBsAg-declining group (Figure 2H). Comparing the mean IL-1β expression levels in the CD14^+^CD16^−^ classical monocyte cluster between the 4 patients with a history of flare and the four without revealed no significant difference, suggesting that elevated IL-1β expression was not associated with flare history (Supplemental Figure S1, http://links.lww.com/HC9/C341). Taken together, these findings suggested that high IL-1β production from the CD14^+^CD16^−^ classical monocytes may be associated with HBsAg decline in CHB patients under NA treatment.

### External validation confirms elevated IL-1β expression in CD14^+^ monocytes of HBsAg-declining CHB patients

To validate our findings, we next prospectively enrolled 32 CHB patients who underwent NA treatment for more than 2 years and showed HBsAg levels higher than 1000 IU/mL or above the upper limit of quantification at the initiation of NA therapy. Among 31 patients, 7 patients had HBsAg levels lower than 100 IU/mL (declining group) and 24 patients had higher than 1000 IU/mL (prolonged group) at enrollment. The clinical characteristics of these patients were shown in Supplemental Table S2, http://links.lww.com/HC9/C340. The patients in the declining group were significantly older than those in the prolonged group. We collected PBMCs from these patients and isolated CD14^+^ monocytes from PBMCs by MACS. The IL-1β mRNA levels were significantly higher in the declining group than those in the prolonged group (Figure 2G), externally validating our finding derived from the scRNA-seq.

### HBsAg-induced IL-1β production in CD14^+^ monocytes is enhanced in HBsAg-declining patients and suppresses HBsAg expression in HBV-producing cells

**FIGURE 3:**
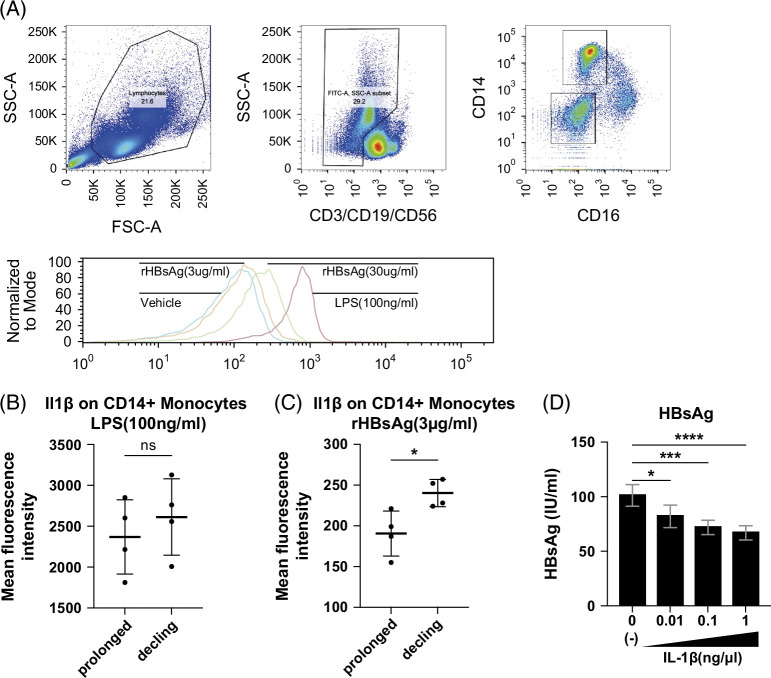
The stronger response of IL-1β production from CD14^+^ monocytes upon HBsAg stimuli is associated with HBsAg decline in CHB patients under NA treatment. (A) Flow cytometric gating strategy to identify the CD14^+^ monocytes. Histogram showing IL-1β expression in CD14^+^ monocytes from healthy controls under the indicated stimulation conditions. (B) Mean fluorescence intensity of IL-1β in CD14^+^ monocytes from declining and prolonged groups under the LPS stimulation. (C) Mean fluorescence intensity of IL-1β in CD14^+^ monocytes from each group under the recombinant HBsAg stimulation. *p<0.05. (D) HBsAg concentration in the supernatant of HepG2.2.15 cells treated with recombinant IL-1β for 17 days at the indicated concentrations. *p<0.05. Abbreviations: CHB, chronic hepatitis B; HBsAg, hepatitis B surface antigen; IL-1β, interleukin-1 beta; LPS, lipopolysaccharide; NA, nucleos(t)ide analogue; rHBsAg, recombinant HBsAg.

To investigate the regulatory mechanisms of IL-1β levels in CD14^+^ monocytes in CHB patients, we treated PBMCs with recombinant HBsAg (rHBsAg) or lipopolysaccharide (LPS). We confirmed that LPS stimulation markedly induced IL-1β production in CD14^+^ monocytes (Figure 3A). Importantly, rHBsAg also induced IL-1β production in CD14^+^ monocytes in a dose-dependent manner (Figure 3A). We then collected PBMCs from declining and prolonged groups and compared the ability of IL-1β production in CD14^+^ monocytes. While LPS stimulation induced IL-1β production in both declining and prolonged groups at similar levels (Figure 3B), the declining groups produced significantly higher IL-1β levels than those in the prolonged groups upon rHBsAg stimulation (Figure 3C). Collectively, our findings suggested that CD14^+^ monocytes from the declining groups may have a greater potential for producing IL-1β upon HBsAg stimuli. Next, to study the causal relationship between IL-1β and HBsAg reduction, we stimulated HBV-producing HepG2.2.15 cells with recombinant IL-1β. Recombinant IL-1β significantly reduced the HBsAg levels of cell-culture supernatant of HBV-producing cells in a concentration-dependent manner (Figure 3D).

### Serum IL-1β level predicts subsequent HBsAg decline in CHB patients undergoing long-term NA therapy

**FIGURE 4:**
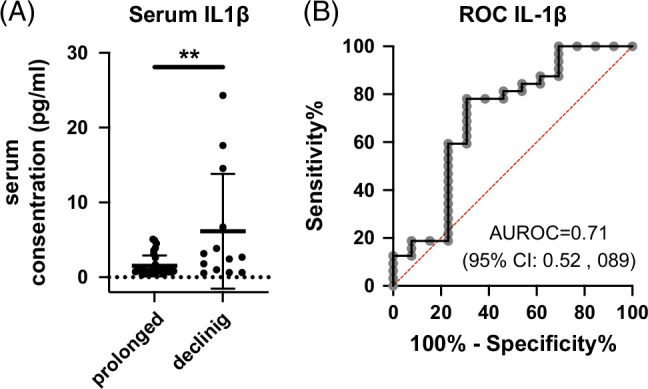
A long-term follow-up cohort revealed the association between serum IL-1β levels and HBsAg decline in CHB patients under NA treatment. (A) The serum IL-1β levels of 45 patients (the declining group: n=13, the prolonged group: n=32) were assessed 1 year after the initiation of NA therapy. (B)The AUROC of the serum IL-1β levels predicting future HBsAg decline. **p<0.01. Abbreviations: AUROC, area under the receiver operating characteristic curve; CHB, chronic hepatitis B; HBsAg, hepatitis B surface antigen; IL-1β, interleukin-1 beta; NA, nucleos(t)ide analogue; ROC, receiver operating characteristic.

Lastly, we evaluated the potential of serum IL-1β levels to predict the future HBsAg decline in CHB patients with NA treatments. To this end, we enrolled 45 patients with chronic hepatitis B (CHB) who had baseline HBsAg levels >1000 IU/mL and whose serum samples were stored within 2 years after initiation of nucleos(t)ide analogue (NA) therapy. We measured serum IL-1β concentrations using the stored samples and compared them between patients who maintained HBsAg >1000 IU/mL (the prolonged group) and those whose HBsAg levels decreased to <100 IU/mL (the declining group) over more than three years of subsequent treatment. The clinical background of these patients is shown in Table 1. The declining group showed a higher proportion of male patients, higher AST and ALT levels, and a longer observational time compared with the prolonged group (Table 1). The serum IL-1β levels were significantly higher in the declining group than in the prolonged group (Figure 4A). The AUROC of the serum IL-1β levels predicting future HBsAg decline was 0.71 with a sensitivity of 0.6923 and specificity of 0.7879 (Figure 4B). The univariate logistic regression analysis revealed that male, high AST levels, high ALT levels, HBeAg negative, long observation period, and high IL-1β were associated with HBsAg decline (Table 2). The multivariate analysis demonstrated that high serum IL-1β levels were independently associated with HBsAg decline in CHB patients who underwent NA treatment (Table 2 and Supplemental Tables S3–S6, http://links.lww.com/HC9/C340). Collectively, these findings suggested that serum IL-1β level was a predictor of subsequent HBsAg decline in NA-treated CHB patients.

**TABLE 1:** Characteristics of HBsAg declining and HBsAg prolonged groups in the long-term follow-up cohort

	HBsAg declining group	HBsAg prolonged group	*p*
Number of patients	13	32	
Age (mean, range)	51.0 (33–69)	49.8 (32–67)	0.7209
Gender (male, female)	10/3	12/20	<0.05
Total bilirubin (mg/dL) (mean, range)	0.8 (0.3–1.3)	0.7 (0.2–1.4)	0.0685
AST (IU/L) (mean, range)	30.2 (16–58)	23.0 (14–42)	<0.05
ALT (IU/L) (mean, range)	30.3 (12–68)	19.7 (8–54)	<0.05
γGTP (IU/L) (mean, range)	31.1 (3–82)	21.9 (10–94)	0.1410
PT (%) (mean, range)	94.3 (60–119)	97.7 (65–121)	0.5090
Albumin (g/dL) (mean, range)	4.2 (3.3–4.7)	4.3 (3.6–4.8)	0.4231
Platelet (×10^3^/μL)	158.3 (48–299)	196.8 (56–305)	0.0550
HBV signal (+, −)	10/3	23/9	>0.9999
HBsAg (IU/mL) (mean, range)	6062 (1270–44,616)	10,211 (1380–52,184)	0.3279
HBeAg (+, −)	2/11	14/18	0.0943
AFP (ng/mL) (mean, range)	1.8 (0.8–4.7)	2.4 (0.8–5.7)	0.1370
NA treatment period prior to sample collection (d) (mean, range)	474.7 (356–701)	442.9 (294–644)	0.2340
Observation period (d) (mean, range)	4542 (10,184–6229)	3575 (1043–6184)	<0.05

Abbreviations: AFP, alpha-fetoprotein; ALT, alanine aminotransferase; AST, aspartate aminotransferase; CHB, chronic hepatitis B; γGTP, gamma-glutamyl transpeptidase; HBeAg, hepatitis B e antigen; HBsAg, hepatitis B surface antigen; HBV, hepatitis B virus; NA, nucleos(t)ide analogue; PT, prothrombin time.

**TABLE 2:** Predictive factors for subsequent HBsAg decline in CHB patients undergoing long-term NA therapy

	Univariate analysis		Multivariate analysis	
	OR (95% CI)	*p*	OR (95% CI)	*p*
Age, years old	1.02 (0.95–1.08)	0.7128		
Gender, M/F	5.56 (1.27–24.29)	0.0145		
Total bilirubin, mg/dL	8.16 (0.80–83.17)	0.0668		
AST, U/L	1.09 (1.01–1.18)	0.0190		
ALT, U/L	1.07 (1.01–1.14)	0.0118	1.07 (1.00–1.15)	0.0367
γGTP, U/L	1.02 (0.99–1.06)	0.1493		
PT, %	0.99 (0.94–1.03)	0.5617		
Alb, g/dL	0.46 (0.07–2.96)	0.4155		
PLT, ×10^3^/μL	0.98 (0.98–1.00)	0.0511		
HBV signal, +/−	1.30 (0.29–5.86)	0.7261		
HBsAg, U/mL	1.00 (1.00–1.00)	0.2698		
HBeAg, +/−	0.20 (0.04–1.08)	0.0388		
AFP, ng/mL	0.62 (0.33–1.17)	0.1117		
NA treatment period, days	1.00 (1.00–1.01)	0.2298		
Observation period, days	1.00 (1.00–1.00)	0.0376		
IL1B, pg/mL	1.41 (1.10–2.17)	0.0017	1.34 (1.07–2.07)	0.0050

Abbreviations: AFP, alpha-fetoprotein; Alb, albumin; ALT, alanine aminotransferase; AST, aspartate aminotransferase; CI, confidence interval; γGTP, gamma-glutamyl transpeptidase; HBeAg, hepatitis B e antigen; HBsAg, hepatitis B surface antigen; HBV, hepatitis B virus; IL1B, interleukin-1 beta; NA, nucleos(t)ide analogue; OR, odds ratio; PLT, platelet; PT, prothrombin time.

## DISCUSSION

In this study, we identified IL-1β as a key immune mediator associated with serum HBsAg decline during NA therapy in patients with CHB. Through integrated single-cell transcriptomics, ex vivo stimulation assays, validation in an independent patient cohort, and long-term clinical follow-up, we demonstrate that elevated IL-1β production by circulating CD14^+^ classical monocytes is associated with the natural reduction of HBsAg levels and may contribute functionally to this process.

Previous studies have highlighted IL-1β as a potent innate immune cytokine capable of restricting HBV replication. In vitro, IL-1β has been shown to suppress HBV expression in infected hepatocytes or HBV-integrated hepatoma cells.^21^,^22^,^23^,^24^ One study further revealed that IL-1β induced antiviral effects via activation of NF-κB pathways but not via induction of interferon-stimulated genes.^22^ Our finding that recombinant IL-1β reduces HBsAg production in HBV-producing HepG2.2.15 cells in a dose-dependent manner is consistent with these prior reports and supports a direct functional role for IL-1β in viral antigen control. In addition, IL-1β may enhance antigen processing and presentation, promote recruitment of effector immune cells, or influence adaptive immunity through modulation of dendritic cell maturation and T-cell priming.^25^ Indeed, our single-cell RNA-seq analysis showed the activation of NK and T cells as well as the upregulation of antigen presentation ability of CD14^+^ monocytes in CHB patients who produced high levels of IL-1β in CD14^+^ monocytes. These findings raise the possibility that augmenting IL-1 signaling may be a viable adjunct to existing or emerging antiviral therapies, aiming for a functional cure.

In CHB, monocyte function is known to be dysregulated. Several studies have reported functional impairments in circulating monocytes, including reduced cytokine secretion, impaired antigen presentation, and altered differentiation potential.^26^ Our single-cell and validation cohort analyses revealed that IL-1β production by CD14^+^CD16^−^ classical monocytes is selectively enhanced in patients with subsequent HBsAg decline, suggesting the existence of functionally preserved or “trained” monocyte subsets capable of mounting effective innate responses to viral antigens in these individuals. Indeed, our ex vivo study revealed the higher capacity of IL-1β production upon HBsAg stimuli in CD14^+^ monocytes of CHB patients with HBsAg reduction compared with those without HBsAg reduction. This may reflect host-intrinsic differences in immune programming or epigenetic reconditioning under prolonged NA suppression. The present study did not elucidate the underlying causes of the differences in IL-1β production capacity. Further research is needed to investigate potential contributing factors, including genetic variations such as single-nucleotide polymorphisms (SNPs),^27^,^28^ as well as epigenetic mechanisms.^29^

Several reports have highlighted the clinical significance of circulating IL-1β levels in patients with HBV infection. Hu et al^24^ reported that in CHB patients treated with combination therapy of pegylated interferon (Peg-IFN) and tenofovir disoproxil fumarate (TDF), plasma IL-1β levels increased in cases that observed reductions in HBV DNA and HBsAg levels. In addition, Kawagishi et al^30^ demonstrated that during direct-acting antiviral (DAA) therapy for chronic hepatitis C, patients who experienced HBV reactivation had lower baseline serum IL-1β levels, and suggested that the IL-1β gene polymorphism rs16944 AA allele may be associated with IL-1β expression and risk of reactivation. These findings suggest that circulating IL-1β may play an important role in the control of HBV and in achieving a functional cure. However, to date, no studies have demonstrated that serum IL-1β levels can predict future HBsAg decline in CHB patients receiving NA therapy. Our results suggest that IL-1β might serve as a useful biomarker for predicting the likelihood of achieving a functional cure. Recently, advances in nucleic acid–based therapeutics, including small interfering RNAs and antisense oligonucleotides, have expanded the landscape of antiviral strategies. A recent study demonstrated that patients who responded immunologically to a therapeutic vaccine achieved markedly higher rates of HBV functional cure when treated with the small interfering RNA elebsiran in combination with pegylated interferon alfa.^30^ Furthermore, previous evidence has shown that genetic polymorphisms associated with IL-1β expression influence the antibody response to hepatitis B vaccination.^31^ Taken together, these findings suggest that biomarkers related to IL-1β expression may prove valuable for guiding future therapeutic decisions.

Nonetheless, several limitations must be acknowledged. The single-cell analysis included a relatively small number of patients and target genes, although the consistency of results across independent validation and longitudinal cohorts mitigates this concern. Functional studies were limited to in vitro models, and in vivo validation in animal models or clinical intervention studies will be necessary to establish causality. Detailed clinical background information, including the history of flare and comorbidities, could not be fully confirmed due to the retrospective design, which is a limitation of the present study. In addition, the absolute differences in IL-1β expression were modest, suggesting that improvements in assay sensitivity may be necessary for clinical application. Moreover, while IL-1β shows promise as a biomarker and immune effector, its proinflammatory nature mandates caution in therapeutic applications to avoid hepatotoxicity. Excessive IL-1β signaling has been associated with hepatic inflammation and injury in experimental models under certain conditions, such as steatohepatitis.^31^,^32^ Therefore, modulation of IL-1β–associated pathways, rather than direct cytokine administration, may represent a more feasible therapeutic strategy. At present, IL-1β should be considered primarily as a marker of effective immune activation, and additional in vivo studies are needed to clarify its therapeutic potential.

## CONCLUSIONS

In conclusion, our study provides novel insights into the role of monocyte-derived IL-1β in the decline of serum HBsAg during NA therapy for CHB. IL-1β not only emerges as a mechanistically relevant biomarker but may also represent a future therapeutic target for strategies aimed at achieving a functional cure.

## Supplementary Material
