# Supplementary material for: Monocyte-derived IL-1β predicts and promotes HBsAg decline in chronic hepatitis B patients under nucleoside analogue therapy
Source: Hepatol Commun. 2026 May 26;10(6):e0957. doi: 10.1097/HC9.0000000000000957 (PMC13209470; doi:10.1097/HC9.0000000000000957)
Supplement: Supplementary file 1 [file hc9-10-e0957-s001.pdf]

Supplementary Table 1

				Before treatment					Treatment		At PBMC collection					
	Case No.	Age	Sex	HBsAg (IU/ml)	HBV-DNA (logIU/ml)	HBeAg/ HBeAb	ALT (U/L)	Prior flare	Drug	Term (year)	HBsAg (IU/ml)	HBeAg/ HBeAb	ALT (U/L)	Diabetes mellitus	Dyslipidemia	Fatty liver
HBsAg declining group	1	47	M	**1853**	7.0	-/+	717	+	ETV	9	**37**	-/+	25	-	-	-
	2	66	M	**2500**	8.4	+/-	54	-	ETV	5	**34**	-/+	15	-	-	-
	3	55	M	**2500≦**	7.1	+/-	47	+	LAM, ETV	14	**100**	-/-	34	-	+	+
	4	61	M	**2500≦**	7.6≦	+/-	152	-	ETV, TAF	14	**62**	-/-	28	-	-	+
mean		57.2		2338.3	7.5		242.5			10.5	58.3		25.5			
HBsAg prolonged group	5	50	M	**1260**	6.9	-/+	374	+	ETV, TAF	7	**3566**	-/+	35	-	+	-
	6	47	M	**10000≦**	9.0≦	+/-	229	+	IFN,ETV,TAF	5	**1666**	+/-	12	-	-	-
	7	47	M	**8307**	7.4	-/+	139	-	ETV,IFN,TAF	10	**1240**	-/+	15	-	-	-
	8	57	F	**2742**	6.8	-/+	51	-	ETV	14	**1048**	-/+	15	-	-	-
mean		50.3		5577.3	7.5		198.3			9.0	1880		19.3			
P value		0.301		0.301	0.686		0.886			0.743	<0.05		0.460			

Supplementary Table 2

	HBsAg declining group	HBsAg prolonged group	p-value	Missing values
Number of patients	7	24		
Age (mean, range)	61.3 (53-69)	49.7 (37-60)	<0.005	0
Gender (male, female)	5 / 2	12 / 12	0.4117	0
Diabetes mellitus (+, −)	0 / 7	1 / 23	>0.999	-
Dyslipidemia (+, −)	3 / 3	3 / 21	0.0754	-
Total bilirubin (mg/dL) (mean, range)	0.8(0.4-0.9)	0.7(0.3-1.1)	0.8173	0
AST (IU/L) (mean, range)	27.3(14-61)	22.0(15-30)	0.1790	0
ALT (IU/L) (mean, range)	28.4(9-78)	22.2(9-58)	0.3089	0
γGTP (IU/L) (mean, range)	72.0(17-299)	33.4(11-328)	0.2267	0
PT(%) (mean, range)	102.7(75-107)	103.7(83-117.6)	0.7941	4
Albumin (g/dL) (mean, range)	4.5(4.3-4.8)	4.4(4.0-4.7)	0.1520	0
Platelet ($\times 10^3/\mu L$)	188.3(128-248)	228.2(137-368)	0.0716	0
HBV signal	0 / 7	3 / 21	>0.999	0
HBsAg at start of treatment (IU/L) (mean, range)	-- #	14447.9	--	
HBeAg (+, −)	0 / 7	8 /16	0.1460	0
AFP (ng/mL)(mean, range)	2.4(2-3)	2.6(1-8)	0.8379	1
Fatty liver (+, −)	2 / 5	4 / 20	0.5959	-
NA treatment period (years)	9.00(6-14)	7.88(2-14)	0.4051	0
Prior flare (+, −)	1 / 6	4 / 20	>0.999	-

Many cases were above the upper limit.

Supplementary Table 3

		Univariate Analysis		Multivariate Analysis	
		OR (95% CI)	*p* - value	OR (95% CI)	*p* - value
Age	years old	1.02(0.95-1.08)	0.7128		
Gender	M/F	5.56(1.27-24.29)	0.0145	12.00(1.99-171.28)	0.0048
Total bilirubin	mg/dL	8.16(0.80-83.17)	0.0668		
AST	U/L	1.09(1.01-1.18)	0.0190		
ALT	U/L	1.07(1.01-1.14)	0.0118		
γGTP	U/L	1.02(0.99-1.06)	0.1493		
PT	%	0.99(0.94-1.03)	0.5617		
Alb	g/dL	0.46(0.07-2.96)	0.4155		
PLT	$\times 10^3/\mu L$	0.98(0.98-1.00)	0.0511		
HBV signal	+/-	1.30(0.29-5.86)	0.7261		
HBsAg	U/mL	1.00(1.00-1.00)	0.2698		
HBeAg	+/-	0.20(0.04-1.08)	0.0388		
AFP	ng/mL	0.62(0.33-1.17)	0.1117		
NA treatment period	days	1.00(1.00-1.01)	0.2298		
Observation period	days	1.00(1.00-1.00)	0.0376		
IL1B	pg/mL	1.41(1.10-2.17)	0.0017	1.44(1.13-2.27)	0.0006

Supplementary Table 4

		Univariate Analysis		Multivariate Analysis	
		OR (95% CI)	*p* - value	OR (95% CI)	*p* - value
Age	years old	1.02(0.95-1.08)	0.7128		
Gender	M/F	5.56(1.27-24.29)	0.0145		
Total bilirubin	mg/dL	8.16(0.80-83.17)	0.0668		
AST	U/L	1.09(1.01-1.18)	0.0190	1.10(1.02-1.22)	0.0179
ALT	U/L	1.07(1.01-1.14)	0.0118		
γGTP	U/L	1.02(0.99-1.06)	0.1493		
PT	%	0.99(0.94-1.03)	0.5617		
Alb	g/dL	0.46(0.07-2.96)	0.4155		
PLT	$\times 10^3/\mu L$	0.98(0.98-1.00)	0.0511		
HBV signal	+/-	1.30(0.29-5.86)	0.7261		
HBsAg	U/mL	1.00(1.00-1.00)	0.2698		
HBeAg	+/-	0.20(0.04-1.08)	0.0388		
AFP	ng/mL	0.62(0.33-1.17)	0.1117		
NA treatment period	days	1.00(1.00-1.01)	0.2298		
Observation period	days	1.00(1.00-1.00)	0.0376		
IL1B	pg/mL	1.41(1.10-2.17)	0.0017	1.47(1.10-2.37)	0.0016

Supplementary Table 5

		Univariate Analysis		Multivariate Analysis	
		OR (95% CI)	p - value	OR (95% CI)	p - value
Age	years old	1.02(0.95-1.08)	0.7128		
Gender	M/F	5.56(1.27-24.29)	0.0145		
Total bilirubin	mg/dL	8.16(0.80-83.17)	0.0668		
AST	U/L	1.09(1.01-1.18)	0.0190		
ALT	U/L	1.07(1.01-1.14)	0.0118		
γGTP	U/L	1.02(0.99-1.06)	0.1493		
PT	%	0.99(0.94-1.03)	0.5617		
Alb	g/dL	0.46(0.07-2.96)	0.4155		
PLT	$\times 10^3/\mu L$	0.98(0.98-1.00)	0.0511		
HBV signal	+/-	1.30(0.29-5.86)	0.7261		
HBsAg	U/mL	1.00(1.00-1.00)	0.2698		
HBeAg	+/-	0.20(0.04-1.08)	0.0388	0.31(0.04-1.54)	0.1578
AFP	ng/mL	0.62(0.33-1.17)	0.1117		
NA treatment period	days	1.00(1.00-1.01)	0.2298		
Observation period	days	1.00(1.00-1.00)	0.0376		
IL1B	pg/mL	1.41(1.10-2.17)	0.0017	1.38(1.06-2.14)	0.0059

Supplementary Table 6

		Univariate Analysis		Multivariate Analysis	
		OR (95% CI)	*p* - value	OR (95% CI)	*p* - value
Age	years old	1.02(0.95-1.08)	0.7128		
Gender	M/F	5.56(1.27-24.29)	0.0145		
Total bilirubin	mg/dL	8.16(0.80-83.17)	0.0668		
AST	U/L	1.09(1.01-1.18)	0.0190		
ALT	U/L	1.07(1.01-1.14)	0.0118		
γGTP	U/L	1.02(0.99-1.06)	0.1493		
PT	%	0.99(0.94-1.03)	0.5617		
Alb	g/dL	0.46(0.07-2.96)	0.4155		
PLT	$\times 10^3/\mu L$	0.98(0.98-1.00)	0.0511		
HBV signal	+/-	1.30(0.29-5.86)	0.7261		
HBsAg	U/mL	1.00(1.00-1.00)	0.2698		
HBeAg	+/-	0.20(0.04-1.08)	0.0388		
AFP	ng/mL	0.62(0.33-1.17)	0.1117		
NA treatment period	days	1.00(1.00-1.01)	0.2298		
Observation period	days	1.00(1.00-1.00)	0.0376	1.00(1.00-1.00)	0.4106
IL1B	pg/mL	1.41(1.10-2.17)	0.0017	1.34(1.05-2.08)	0.0127
