# Supplementary material for: Monocyte-derived IL-1β predicts and promotes HBsAg decline in chronic hepatitis B patients under nucleoside analogue therapy
Source: Hepatol Commun. 2026 May 26;10(6):e0957. doi: 10.1097/HC9.0000000000000957 (PMC13209470; doi:10.1097/HC9.0000000000000957)
Supplement: Supplementary file 2 [file hc9-10-e0957-s002.pdf]

Supplementary Fig.1

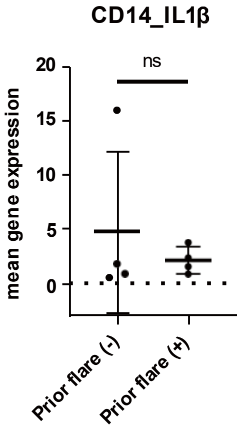

Supple Fig.1

IL-1β expression levels in the $CD14^{+}CD16^{-}$ classical monocyte cluster of peripheral blood mononuclear cells from patients with CHB, analyzed by scRNA-seq in the indicated groups (n = 4 each for prior flare (−) and (+)).
